# Sequence of COVID-19 Vaccination and COVID-19 Infection and Their Association With the Development of Active Tuberculosis: A Case-Control Study

**DOI:** 10.7759/cureus.46353

**Published:** 2023-10-02

**Authors:** Satiti Palupi, Imran Pambudi, Asik Surya, Rendra Bramanthi, Mohamad Arfi, Suyanto Suyanto, Kyaw Ko Ko Htet, Virasakdi Chongsuvivatwong

**Affiliations:** 1 Department of Epidemiology, Prince of Songkla University, Hat Yai, THA; 2 Directorate of Direct Communicable Disease Prevention and Control, Ministry of Health Republic of Indonesia, Jakarta, IDN; 3 Department of Microbiology, RSUD (Rumah Sakit Umum Daerah) Dr. Iskak Hospital, Tulungagung, IDN; 4 Department of Pulmonology, RSUD (Rumah Sakit Umum Daerah) Dr. Iskak Hospital, Tulungagung, IDN; 5 Faculty of Medicine, Riau University, Pekanbaru, IDN

**Keywords:** indonesia, case-control study, active tuberculosis, covid-19 vaccination, covid-19 infection

## Abstract

Introduction

Information regarding the cross-risk of coronavirus disease 2019 (COVID-19) and tuberculosis (TB) is still sparse. This study aimed to identify the patterns of sequence of COVID-19 vaccination and COVID-19 infection and to explore the association between COVID-19 vaccination, COVID-19 infection, and the development of active TB.

Methods

It was a case-control study conducted in RSUD Dr. Iskak Hospital, Tulungagung, between October 2022 and April 2023. Active cases of TB patients were compared with non-TB controls in the same hospital, with the same age and sex. Their pattern of sequence of COVID-19 vaccination and infection was investigated. Logistic regression was used to assess the association between these key variables.

Results

Of 296 case-control sets, 64.2% were female. The mean ± standard deviation of age was 46 ± 15.6 years. 5.7% of the cases and 6.4% of the controls had a history of COVID-19 infection, whereas 58.8% and 68.4% had been vaccinated (mostly after infection). The adjusted odds ratio (95% confidence interval) of COVID-19 infection on risk to the development of active TB was 1.45 (0.58, 3.65). Those of COVID-19 vaccination of one to four doses were 0.42 (0.17, 1), 0.98 (0.58, 1.66), 0.48 (0.25, 0.93), and 0.09 (0.01, 0.81), respectively.

Conclusion

It was found that there were five patterns of sequence of COVID-19 infection and COVID-19 vaccination, with the most frequent being having COVID-19 infection before COVID-19 vaccination. Our data did not support the association between COVID-19 infection and the subsequent development of active TB. On the other hand, COVID-19 vaccination has been demonstrated to increase some protection against the development of active TB.

## Introduction

Coronavirus disease 2019 (COVID-19) and tuberculosis (TB) are highly transmissible diseases and pose a serious risk to public health [1]. Both diseases present respiratory symptoms and are transmitted through a similar route. The immunological mechanism of co-infection has been identified as a common dysregulation of the immune responses in both COVID-19 and TB [2]. This indicates a dual risk of co-infection and, therefore, requires immediate attention [1]. A previous rapid systematic review and meta‐analysis study showed that the prevalence of TB among COVID-19 patients in the included studies varied from 0.37% to 4.47% [3]. Another systematic review and meta‐analysis found that 0.99% of COVID-19 patients had active pulmonary TB [4]. The study conducted in Cameroon showed that the prevalence of SARS-CoV-2 RNA in pulmonary TB patients was 24.3% [5]. However, information on cross-risk between the two diseases is still sparse. Moreover, COVID-19 vaccinations were given extensively. There is a need to examine the effects of these vaccinations on not only COVID-19 infection but also the risk of development of active TB. 

Based on WHO estimation, Indonesia was ranked as the second country with the highest TB burden. In 2021, the TB incidence rate was 354 per 100,000 population [6]. From the national registry system website of TB, namely Sistem Informasi Tuberculosis (SITB), on July 29th, 2022, in East Java Province, TB cases in 2021 were 42,476. Meanwhile, in Tulungagung district, the total number of cases was 773 [7,8].

On the other hand, based on the national registry system website for COVID-19, namely New All Record (NAR), on July 29th, 2022, the number of COVID-19 cases in Indonesia in 2021 was 3,519,522. Meanwhile, in East Java Province, there were 315,914 cases. Tulungagung district accounted for 8,701 cases [9,10].

In the context of establishing a TB diagnosis in Tulungagung district, approximately 70% were examined at RSUD Dr. Iskak Hospital [7,8], which is a public and class B teaching hospital, and also became a referral hospital for multidrug-resistant TB, owned by the Tulungagung government. For COVID-19, RSUD Dr. Iskak Hospital is “the backbone” of the test in Tulungagung using real-time polymerase chain reaction (RT-PCR). Overall, because of the achievements and good service governance, the Minister of Health, Republic of Indonesia, appreciated RSUD Dr. Iskak Hospital and was interested in making it a role model at the national level.

This was the first study regarding the association between COVID-19 and TB using a hospital-based case-control study. The objective of this study was to identify the patterns of sequence of COVID-19 vaccination and COVID-19 infection and to explore the association between COVID-19 vaccination, COVID-19 infection, and the development of active TB.

## Materials and methods

Study design and population

A case-control study was carried out to assess the development of active TB, and cases were active TB, defined as a newly diagnosed TB patient; classification based on the anatomical location of the disease was pulmonary TB and GeneXpert MTB/RIF result as bacteriologically confirmed in RSUD Dr. Iskak Hospital. For concise communication, we will use active TB throughout the remaining part of this manuscript, while controls were sex- and age-matched attendees who presented to the same hospital for non-TB health problems. Based on the national TB data on July 29th, 2022, the number of bacteriologically confirmed cases of pulmonary TB in East Java Province in 2021 was 51.37%. Meanwhile, the number of bacteriologically confirmed cases of pulmonary TB in Tulungagung district was 35.70%. The rest were clinically diagnosed TB and extrapulmonary TB cases [7,8].

Sample size

**Figure 1:**
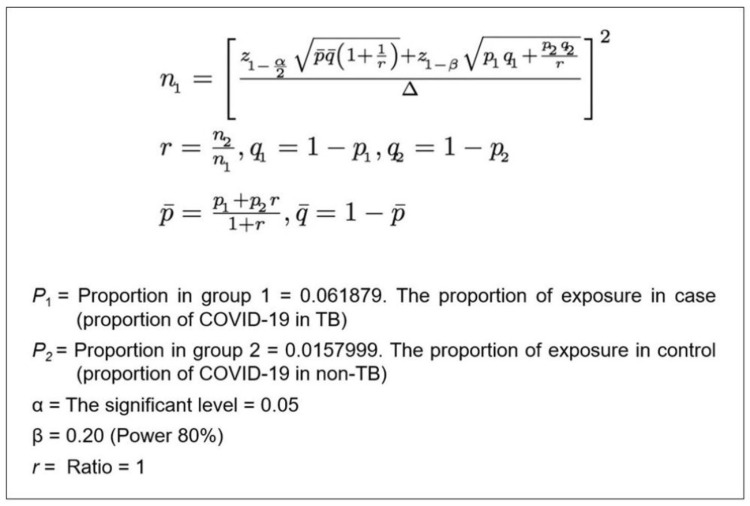
Sample size formula

Data were collected from the registration system's website, namely, SITB for TB and NAR for COVID-19, developed by the Ministry of Health, Republic of Indonesia. This study used a sample size application called “n4Studies” to determine the sample size [11]. This application provided several functions for sample size and power calculations, including calculating sample size for matching the case-control. The formula is shown in Figure 1.

The sample size of the cases was 275. As the ratio of the cases to the controls was decided to be one-to-one, a similar number of controls were also planned to be included in the study, which made the total sample size 550. The proportion of cases and controls in this study was 1:1 because statistical power for testing the null hypothesis would be maximized with the existing limited budget [12].

Sampling procedure

Selection of Cases

The cases were active TB, aged ≥18 years. Subjects were approached when they already knew the results of the diagnosis. The subjects agreed and signed an informed consent. If they refused to be involved in the study, the next eligible subjects were approached. The cases were screened and tested for COVID-19 using Antigen Test Kit (ATK) to exclude COVID-19 infection. The data collection took place until the required sample size was reached. We collected samples beyond the target of sample size because there was still time available for data collection.

Selection of Controls

The subjects attended general checkup or surgery or dental clinic in RSUD Dr. Iskak Hospital, Tulungagung. The attendees who had sex and age groups matched with the respective cases were selected as controls. Subjects who fitted the sex and age group criteria were approached on a clinic day. Subjects agreed and signed an informed consent. If they refused to be involved in the study, the next eligible subjects were approached. The controls were screened for TB and COVID-19 by the hospital health officer, and the subjects were excluded if they were susceptible to TB. Subjects were screened and tested for COVID-19 using ATK to exclude COVID-19 infection. Then, the subjects were examined by chest X-ray, and the results were read by the experts to exclude susceptible findings of TB.

The inclusion and exclusion criteria are summarized in Table 1.

**Table 1:** Inclusion and exclusion criteria for the study

Criteria	Case	Control
Inclusion criteria		
Tuberculosis	Active tuberculosis	Tuberculosis excluded
Age	≥18 years	Matched with the case
Sex	Male, female	Matched with the case
Exclusion criteria		
COVID-19	Current COVID-19	Current COVID-19
Pregnancy	Excluded	Excluded
Tuberculosis status	Relapse tuberculosis	Not available
	Retreatment tuberculosis	Not available
	Extrapulmonary tuberculosis	Not available
	Clinically diagnosed tuberculosis	Not available

Data collection

Participants who met the inclusion criteria and had signed informed consent were directly interviewed by one member of the enumerator team with a well-prepared structured questionnaire. Variables collected included sociodemographic characteristics (sex, age, religion, place of residence, occupation, income, marital status, education, and household members), COVID-19 infection (time of COVID-19 infection, hospitalization), COVID-19 vaccination (time of COVID-19 vaccination, type of vaccination), medical history (diabetes mellitus, hypertension, HIV, asthma), BCG vaccination, and smoking behavior. We also checked the information history of COVID-19 infection and COVID-19 vaccination with the electronic data on the register.

The interview was conducted in an open premise, away from traffic and crowds, to prevent any possible infection from the interview.

Data analysis

The data were collected and entered into the computer using EpiData software version 3.1 (The EpiData Association, Odense, Denmark). Statistical analyses were performed using R version 4.0.2 (R Foundation for Statistical Computing, Vienna, Austria). Descriptive statistics were presented for an initial comparison of baseline characteristics between the cases and the controls. The univariate analysis was performed to examine the effect of each independent variable on the development of active TB.

Logistic regression was constructed, including variables that showed an effect in the prediction of the development of active TB in the univariate analysis with a P-value <0.1. We used a cutoff P-value <0.1 in the univariate analysis to find the independent risk factor candidates.

Based on the principle of directed acyclic graph [13], we tested both the direct effect and the mediated effect association between COVID-19 infection, COVID-19 vaccination, and the development of active TB. For the direct effect of the vaccine, we included the history of COVID-19 infection and prior vaccination in the model. Whether COVID-19 infection was a possible mediator was tested by another model without the vaccine included. Odds ratios (OR), adjusted odds ratios (aOR), and 95% confidence interval (95% CI) were estimated. Following the common practice in statistical analysis, a P-value <0.05 was considered statistically significant.

To identify the patterns of the sequence of COVID-19 vaccination and COVID-19 infection, we sequenced them based on the time of COVID-19 infection and COVID-19 vaccination. Then we divided them into five pattern sequence groups. The R packages we used were epicalc, tidyverse, and lubridate.

Time frame

The study commenced on October 2022 and the data were collected from this period until April 2023. This was a period when the COVID-19 pandemic started to subside, making data collection possible.

## Results

**Figure 2:**
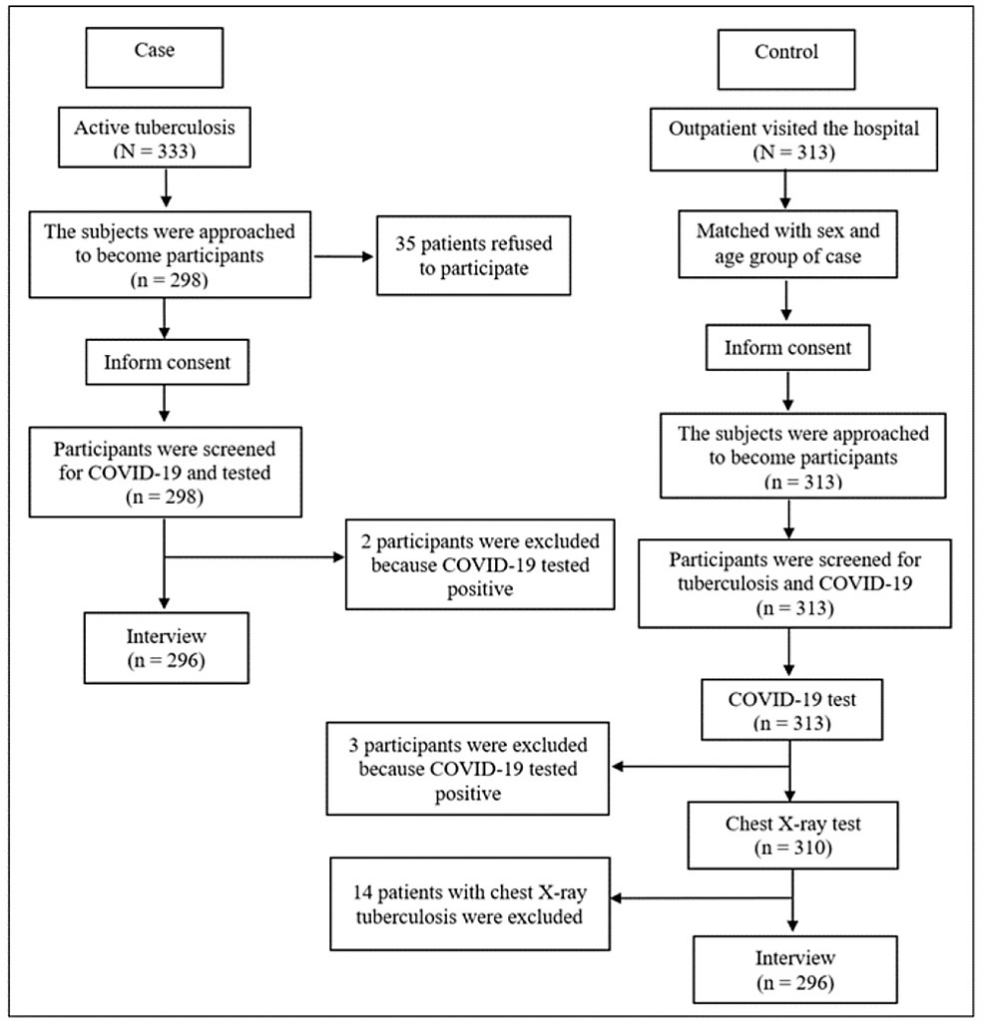
Identification of case and control participants

Figure 2 illustrates the number of subjects recruited, consented, and excluded at each step of the research project. Altogether, the consent rates were high (298 of 333 cases and all the controls). Five subjects were further excluded because of having positive COVID-19 tests, and 14 controls were excluded due to chest X-ray suspicious of pulmonary TB. The subjects were approached to become a participant.

Comparison of characteristics of the cases and the controls 

Table 2 shows that the sex and age groups of the cases and controls were well-balanced. Of 296 case-control sets, 64.2% were female. The mean ± standard deviation of age was 46 ± 15.6 years. 5.7% of the cases and 6.4% of the controls had a history of COVID-19 infection, whereas 58.8% and 68.4% had been vaccinated (mostly after infection). Both groups were mainly Islam, urban residents, employed, and married. The control group had significantly higher levels of income and education than the cases.

**Table 2:** Baseline characteristics of the cases and the controls

Variable	Case n (%)	Control n (%)	Total n (%)	P-value
Total	296	296	592	
Sociodemographic variables				
Sex				
Male	190 (64.2)	190 (64.2)	380 (64.2)	1
Female	106 (35.8)	106 (35.8)	212 (35.8)	
Age group				
≤24	34 (11.5)	34 (11.5)	68 (11.5)	1
25-34	46 (15.5)	46 (15.5)	92 (15.5)	
35-44	41 (13.9)	41 (13.9)	82 (13.9)	
45-54	78 (26.4)	78 (26.4)	156 (26.4)	
55-64	67 (22.6)	67 (22.6)	134 (22.6)	
≥65	30 (10.1)	30 (10.1)	60 (10.1)	
Religion				
Islam	289 (97.6)	295 (99.7)	584 (98.6)	0.068
Christian	7 (2.4)	1 (0.3)	8 (1.4)	
Place of residence				
Rural	46 (15.5)	65 (22)	111 (18.8)	0.058
Urban	250 (84.5)	231 (78)	481 (81.2)	
Occupation				
Unemployed	102 (34.5)	80 (27)	182 (30.7)	0.061
Employed	194 (65.5)	216 (73)	410 (69.3)	
Income per month				
Low income	142 (70.3)	83 (37.9)	225 (53.4)	<0.001
High income	60 (29.7)	136 (62.1)	196 (46.6)	
Marital status				
Married	201 (67.9)	219 (74)	420 (70.9)	0.05
Separate	40 (13.5)	22 (7.4)	62 (10.5)	
Single	55 (18.6)	55 (18.6)	110 (18.6)	
Education				
Low education	156 (52.7)	94 (31.8)	250 (42.2)	<0.001
Middle education	112 (37.8)	140 (47.3)	252 (42.6)	
High education	28 (9.5)	62 (20.9)	90 (15.2)	
Household member				
≤4 members	208 (70.3)	206 (69.6)	414 (69.9)	0.929
>4 members	88 (29.7)	90 (30.4)	178 (30.1)	
Underlying conditions				
BCG history				
No	21 (7.1)	29 (9.8)	50 (8.4)	<0.001
Yes	204 (68.9)	117 (39.5)	321 (54.2)	
Unknown	71 (24)	150 (50.7)	221 (37.3)	
Smoking within 30 days				
No	226 (76.4)	142 (48)	368 (62.2)	<0.001
Yes	70 (23.6)	154 (52)	224 (37.8)	
Number of cigarettes per day				
1-10	34 (48.6)	99 (64.3)	133 (59.4)	<0.001
11-20 cigarettes per day	31 (44.3)	55 (35.7)	86 (38.4)	
>20 cigarettes per day	5 (7.1)	0 (0)	5 (2.2)	
Diabetes mellitus				
No	231 (78)	240 (81.1)	471 (79.6)	0.415
Yes	65 (22)	56 (18.9)	121 (20.4)	
Hypertension				
No	268 (90.5)	236 (79.7)	504 (85.1)	<0.001
Yes	28 (9.5)	60 (20.3)	88 (14.9)	
HIV				
No	289 (97.6)	296 (100)	585 (98.8)	0.015
Yes	7 (2.4)	0 (0)	7 (1.2)	
Asthma				
No	288 (97.3)	295 (99.7)	583 (98.5)	0.038
Yes	8 (2.7)	1 (0.3)	9 (1.5)	

There was a large proportion of unknown BCG history. Smoking within 30 days and hypertension were associated with a lower risk of TB. We consider that these were reverse causal relationships and, therefore, these variables were not included as predictors. The number of HIV-positive and asthma cases was low. These two variables were also not included because they would have caused instability in the model.

Bacteriological information of the cases

Appendix Table 6 shows the cross-distribution of levels of *Mycobacterium tuberculosis *(*M. tb*) detected and rifampicin resistance of the cases. There were 20 (6.8%) rifampicin resistance patients. The majority had a medium level of *M. tb.*

The main health problem of control participants visiting the hospital

Table 3 shows that most of the control participants visited the General Checkup clinic (52%). The main reason was for checkups (91.6%). Chest X-ray results were normal in 74.3%. The remaining had respiratory and other problems.

**Table 3:** Clinics visited, the main reason for admission, and chest X-ray result of the control participants at the hospital

Variable	n (%)
The clinic visited at the hospital	
General checkup clinic	154 (52)
Surgery clinic	139 (47)
Dental clinic	3 (1)
The main reason for admission to the hospital	
Checkup	272 (91.9)
Surgery	16 (5.4)
Pain	4 (1.3)
Fall control	2 (0.7)
Difficulty in urination	2 (0.7)
Chest X-ray results	
Normal	220 (74.3)
Pneumonia	32 (10.8)
Heart disease	24 (8.1)
Emphysema	9 (3.1)
Mass	6 (2.0)
Others	5 (1.7)

COVID-19 infection and COVID-19 vaccination history

From Table 4, it can be seen that there were 17 cases and 19 controls having COVID-19 infection. Five cases and six controls needed hospitalization. None got pneumonia. So, most were mild forms of infection. These relatively small numbers suggested that our subjects did not commonly suffer from COVID-19. The difference in COVID-19 infection between these two groups was not significant. There were five patterns of sequence of COVID-19 vaccination and COVID-19 infection. The most common was having COVID-19 infection before COVID-19 vaccination.

**Table 4:** History of COVID-19 infection and vaccination between the cases and the controls

Variable	Case n (%)	Control n (%)	Total n (%)	P-value
Total	296	296	592	
COVID-19 Infection				
No	190 (64.2)	277 (93.6)	556 (93.9)	0.863
Yes	106 (35.8)	19 (6.4)	36 (6.1)	
Patterns of Sequence of COVID-19 Vaccination and COVID-19 Infection				
COVID-19 infection; vaccinations	8	5		
COVID-19 infection; no vaccination	4	3		
1st Vaccination; COVID-19 infection; 2nd Vaccination	3	4		
1st Vaccination; 2nd vaccination; COVID-19 infection; 3rd vaccination	2	5		
1st Vaccination; 2nd vaccination; 3rd vaccination; COVID-19 infection	0	2		
COVID-19 Vaccination				
None	99 (33.4)	67 (22.6)	166 (28)	<0.001
One dose	19 (6.4)	25 (8.4)	44 (7.4)	
Two doses	130 (43.9)	102 (34.5)	232 (39.2)	
Three doses	47 (15.9)	91 (30.7)	138 (23.3)	
Four doses	1 (0.3)	11 (3.7)	12 (2)	

Most participants had been COVID-19 vaccinated, and the majority (52%) received two doses. Most of the first and the second vaccines (37.8% and 34%) were CoronaVac, and the third was Pfizer-BioNTech (11.1%).

A comparison of the number of doses of COVID-19 vaccination among cases and controls was done. A higher percentage of the cases (33.4%) than the controls (22.6%) had no COVID-19 vaccination history. The overall number of COVID-19 vaccinations was lower in cases (mean [standard deviation] = 1.43 [1.12]) than in controls (mean [standard deviation] = 1.84 [1.19]). The t-test P-value was <0.001.

Multivariate analysis

Table 4 summarizes the results of the logistic regression predicting the development of active TB from various independent variables. The main variable, COVID-19 infection, was not statistically significant, having an aOR of 1.45 and 95% CI of 0.58 to 3.65. On the other hand, COVID-19 vaccination had a significant independent protective effect against the development of active TB (aOR range 0.09 to 0.98, likelihood ratio [LR] test P-value = 0.005). Other significant risk factors included unemployment, low income, and low education levels.

Analysis of effects of COVID-19 vaccination

The effect of COVID-19 vaccination was independent of COVID-19 infection, as seen in Table 5. This can be considered as a “direct” COVID-19 vaccination effect. However, COVID-19 vaccination was given to prevent COVID-19 infection, and COVID-19 infection might affect the development of active TB in our hypothesis. So COVID-19 infection can be a mediator in the effect of COVID-19 vaccination on the development of active TB. Another logistic regression is needed to test the mediator.

**Table 5:** Logistic regression analysis of the association between a COVID-19 infection and the development of active tuberculosis (effect of COVID-19 vaccination)

Variable	Odds ratio (95% confidence interval)	Adjusted odds ratio (95% confidence interval)	P-value
COVID-19 infection			
No	Referent	Referent	0.429
Yes	0.80 (0.37, 1.74)	1.45 (0.58, 3.65)	
COVID-19 vaccination			
None	Referent	Referent	0.005
One dose	0.47 (0.21, 1.06)	0.42 (0.17, 1)	
Two doses	0.86 (0.53, 1.40)	0.98 (0.58, 1.66)	
Three doses	0.32 (0.18, 0.56)	0.48 (0.25, 0.93)	
Four doses	0.06 (0.01, 0.51)	0.09 (0.01, 0.81)	
Religion			
Islam	Referent	Referent	0.021
Christian	0.18 (0.02, 1.56)	0.09 (0.01, 0.95)	
Place of residence			
Rural	Referent	Referent	0.789
Urban	1.18 (0.73, 1.9)	1.08 (0.63, 1.83)	
Occupation			
Unemployed	Referent	Referent	0.030
Employed	0.27 (0.07, 0.98)	0.23 (0.06, 0.97)	
Income			
Low income	Referent	Referent	<0.001
High income	0.26 (0.17, 0.39)	0.32 (0.20, 0.50)	
Marital status			
Married	Referent	Referent	0.311
Separate	1.51 (0.79, 2.91)	1.51 (0.55, 2.39)	
Single	1.52 (0.83, 2.80)	1.72 (0.85, 3.50)	
Education			
Low education	Referent	Referent	0.046
Middle education	0.58 (0.38, 0.89)	0.57 (0.35, 0.94)	
High education	0.25 (0.13, 0.45)	0.45 (0.21, 0.98)	
Household member			
≤4 members	Referent	Referent	0.181
>4 members	1.07 (0.71, 1.63)	1.39 (0.86, 2.24)	
Diabetes mellitus			
No	Referent	Referent	0.592
Yes	1.01 (0.63, 1.63)	0.86 (0.49, 1.49)	

To check whether the mediated pathway could be possible, we reran logistic regression omitting the COVID-19 vaccination variable (see Appendix Table 7). The aOR of COVID-19 infection was 1.14 (95% CI = 0.47, 2.73) and the P-value was 0.773. As COVID-19 infection was not a significant risk factor, it cannot be the mediator.

We reran logistic regression for the total effect by omitting the COVID-19 infection variable. The aORs for the various doses of COVID-19 vaccination were very similar to those in Table 5. The direct effect aORs (95% CI) for one, two, three, and four doses were 0.42 (0.17, 1), 0.98 (0.58, 1.66), 0.49 (0.25, 0.94), and 0.10 (0.01, 0.89), respectively. The P-value from the LR test was 0.006. In summary, the COVID-19 vaccination dose is negatively associated with the risk for the development of active TB, whereas there was no evidence of the association between COVID-19 infection and the development of active TB.

**Figure 3:**
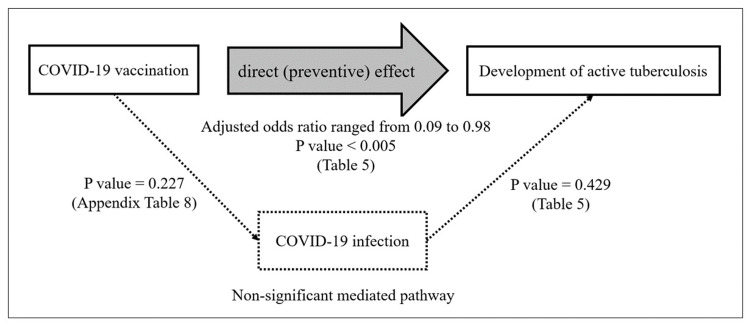
Large direct preventive effect of COVID-19 vaccination on the development of active tuberculosis and no significant mediator role of COVID-19 infection

Figure 3 summarizes the causation flow of COVID-19 vaccination having no significant association with COVID-19 infection (P-value = 0.227) (see Appendix Table 8). COVID-19 vaccination had a strong direct protective effect against the development of active TB and not the intermediary effect of COVID-19 infection.

## Discussion

Our case-control study could achieve the number of cases and controls as required. However, we had to exclude a few subjects among the controls who were not detected with TB. The cases were from lower socioeconomic groups than the controls and were in a worse current health condition. There was no significant difference in the COVID-19 infection but the controls had a high number of doses of COVID-19 vaccination in the past. These results are consistent through all versions of modeling.

While we were conducting this study, COVID-19 infection did not completely disappear as we found few of them in our potential cases and potential controls. COVID-19 infection can take place both before and after COVID-19 vaccination. Similar to other findings in low- and middle-income countries, in our study, it is clear that the exposure variable COVID-19 infection mostly took place before COVID-19 vaccination [14-18].

There were many case-control studies with TB as the main outcome [19-21]. Some of them did not check to make sure that the controls may also have pulmonary TB. So, the study could suffer from the misclassification of the outcome [22,23]. Our study used chest X-rays to exclude the control, which would otherwise be misclassified.

Socioeconomic factors can influence the development of the disease. In our study, unemployment, low income, and low education levels were significant risk factors for the development of active TB. Most cases of TB are found in low-middle-income and low-income countries in the regions of Asia, Africa, and the Western Pacific. The incidence of TB is lower in higher-income and upper-middle-income countries, mostly in Europe and America regions [6,24,25]. Such a difference in prevalence is largely attributed to socioeconomic factors. Low education, unemployment, and low income are the identified social determinants of TB [26-29]. A study in Otwock, Poland and Orel, Russia also found that unemployed workers were more likely to report chronic symptoms of TB; unemployment was related to poverty, and hence the risk of TB [30,31]. Also, the financial impact of TB can be devastating for patients. Loss of income is usually the biggest worry for them [32].

We identified that COVID-19 vaccination has a protective direct effect on decreasing the risk of the development of active TB. The mechanism of the interaction is not yet clear.

The use of inactive vaccines does not affect the results of TB infection tests [33]. On the other hand, vaccination with live viruses, including the measles, mumps, and rubella (MMR) vaccine, can lead to a mild suppression of the immune system and may reduce the reactivity of the TB infection test. However, there is no definitive information on the potential effects of mRNA vaccine on immune responses, and it is not yet known to what extent the COVID-19 mRNA vaccine may have a potential impact on the results of TB infection test during the initial four weeks post-vaccination [34].

Inactivated vaccines, which included CoronaVac, can potentially stimulate both specific adaptive immune responses and non-specific innate immune responses [35-37]. CoronaVac has been shown to stimulate innate immunity in mice, rats, and macaques [38-42]. Transcriptomic overlapping of host to COVID-19 and TB may also partially explain our finding [43]. The study findings may explain our finding on the dose-response relationship between COVID-19 vaccination and the risk for the development of active TB. The implication of this finding included further research to explain this preventive mechanism, which may lead to improvement of the TB vaccine in the future.

In our study, we found that COVID-19 infection has no association with the risk of the development of active TB. This was opposite from the previous report that COVID-19 pneumonia was strongly associated with a higher risk of detectable active pulmonary TB (hazard ratio [95% CI] = 7.15 [5.54, 9.22]) [44]. The two findings had different degrees of severity of COVID-19 infection. In the aforementioned study, all COVID-19 cases used as a cohort were COVID-19 pneumonia cases. COVID-19 pneumonia was associated with weak protective immunity among high-risk subjects [45,46], whereas COVID-19 infection in our study was mostly in a mild form.

The limitation of this study included that other forms of TB, such as latent tuberculosis infection, could not be ruled out. The study was conducted only in one referral hospital. Our hospital-based controls may not fully represent non-TB in the general population, especially in terms of COVID-19 infection history.

## Conclusions

It was found that there were five patterns of sequence of COVID-19 infection and COVID-19 vaccination, with the most frequent being having COVID-19 infection before COVID-19 vaccination. Our data did not support the association between COVID-19 infection and the subsequent development of active TB. On the other hand, COVID-19 vaccination has been demonstrated to increase some protection against the development of active TB.

## Appendices

**Table 6:** Types of tuberculosis drug resistance of case participants

Level of *Mycobacterium tuberculosis* detected	Rifampicin resistance	
	Yes n (%)	No n (%)
High	4 (2.0)	46 (15.5)
Medium	9 (3.1)	118 (39.9)
Low	4 (1.4)	72 (24.3)
Very low	3 (0.3)	40 (13.5)
Total	20 (6.8)	276 (93.2)

**Table 7:** Logistic regression analysis of the association between COVID-19 infection and the development of active tuberculosis (effect of COVID-19 infection)

Variable	Odds ratio (95% confidence interval)	Adjusted odds ratio (95% confidence interval)	P-value
COVID-19 infection			
No	Referent	Referent	0.773
Yes	0.80 (0.37, 1.74)	1.14 (0.47, 2.73)	
Religion			
Islam	Referent	Referent	0.027
Christian	0.18 (0.02, 1.56)	0.11 (0.01, 1.06)	
Place of residence			
Rural	Referent	Referent	0.707
Urban	1.18 (0.73, 1.9)	1.11 (0.65, 1.86)	
Occupation			
Unemployed	Referent	Referent	0.045
Employed	0.27 (0.07, 0.98)	0.27 (0.07, 1.07)	
Income			
Low income	Referent	Referent	<0.001
High income	0.26 (0.17, 0.39)	0.31 (0.20, 0.49)	
Marital status			
Married	Referent	Referent	0.302
Separate	1.51 (0.79, 2.91)	1.21 (0.59, 2.49)	
Single	1.52 (0.83, 2.80)	1.70 (0.85, 3.40)	
Education			
Low education	Referent	Referent	0.003
Middle education	0.58 (0.38, 0.89)	0.56 (0.34, 0.90)	
High education	0.25 (0.13, 0.45)	0.31 (0.15, 0.63)	
Household member			
≤4 members	Referent	Referent	0.179
>4 members	1.07 (0.71, 1.63)	1.38 (0.86, 2.20)	
Diabetes mellitus			
No	Referent	Referent	0.543
Yes	1.01 (0.63, 1.63)	0.84 (0.49, 1.46)	

**Table 8:** Logistic regression analysis of the association between COVID-19 vaccination and COVID-19 infection

Variable	Odds ratio (95% confidence interval)	Adjusted odds ratio (95% confidence interval)	P-value
COVID-19 vaccination			
None	Referent	Referent	0.227
One dose	0.75 (0.08, 6.69)	0.90 (0.10, 8.22)	
Two doses	1.13 (0.36, 3.53)	1.09 (0.33, 3.61)	
Three doses	2.11 (0.68, 6.52)	1.49 (0.39, 5.68)	
Four doses	15.57 (3.63, 66.8)	6.96 (1.20, 40.27)	
Place of residence			
Rural	Referent	Referent	0.900
Urban	1.18 (0.43, 3.19)	0.93 (0.33, 2.68)	
Income			
Low income	Referent	Referent	0.908
High income	1.58 (0.73, 3.42)	0.95 (0.38, 2.38)	
Marital status			
Married	Referent	Referent	0.840
Separate	0.66 (0.15, 2.89)	0.99 (0.21, 4.69)	
Single	0.56 (0.13, 2.44)	0.64 (0.14, 3.03)	
Education			
Low education	Referent	Referent	0.163
Middle education	0.75 (0.27, 2.05)	0.83 (0.27, 2.52)	
High education	3.62 (1.45, 9.03)	2.62 (0.68, 10.09)	
Household member			
≤4 members	Referent	Referent	0.035
>4 members	2.86 (1.32, 6.2)	2.48 (1.07, 5.73)	
Diabetes mellitus			
No	Referent	Referent	0.142
Yes	1.59 (0.68, 3.74)	2.14 (0.79, 5.78)	

**Table 9:** Logistic regression analysis of the association between COVID-19 vaccination and the development of active tuberculosis (effect of COVID-19 vaccination)

Variable	Odds ratio (95% confidence Interval)	Adjusted odds ratio (95% confidence interval)	P-value
COVID-19 vaccination			
None	Referent	Referent	0.006
One dose	0.47 (0.21, 1.06)	0.42 (0.17, 1)	
Two doses	0.86 (0.53, 1.40)	0.98 (0.58, 1.66)	
Three doses	0.32 (0.18, 0.56)	0.49 (0.25, 0.94)	
Four doses	0.06 (0.01, 0.51)	0.10 (0.01, 0.89)	
Religion			
Islam	Referent	Referent	0.023
Christian	0.18 (0.02, 1.56)	0.10 (0.01, 0.98)	
Place of residence			
Rural	Referent	Referent	0.791
Urban	1.18 (0.73, 1.9)	1.07 (0.63, 1.83)	
Occupation			
Unemployed	Referent	Referent	0.034
Employed	0.27 (0.07, 0.98)	0.24 (0.06, 0.99)	
Income			
Low income	Referent	Referent	<0.001
High income	0.26 (0.17, 0.39)	0.32 (0.20, 0.50)	
Marital status			
Married	Referent	Referent	0.318
Separate	1.51 (0.79, 2.91)	1.15 (0.55, 2.40)	
Single	1.52 (0.83, 2.80)	1.71 (0.85, 3.47)	
Education			
Low education	Referent	Referent	0.049
Middle education	0.58 (0.38, 0.89)	0.57 (0.35, 0.94)	
High education	0.25 (0.13, 0.45)	0.46 (0.21, 1)	
Household member			
≤4 members	Referent	Referent	0.153
>4 members	1.07 (0.71, 1.63)	1.41 (0.88, 2.28)	
Diabetes mellitus			
No	Referent	Referent	0.623
Yes	1.01 (0.63, 1.63)	0.87 (0.50, 1.51)	
